# Idiosyncratic Drug Reaction: A Rare Mechanism of Acute Tylenol Toxicity

**DOI:** 10.7759/cureus.6099

**Published:** 2019-11-08

**Authors:** Ahmad Raza, Vincent Chan, Muhammad Umair Atiq

**Affiliations:** 1 Internal Medicine, Abington Hospital-Jefferson Health, Abington, USA

**Keywords:** tylenol, liver failure, idiosyncratic

## Abstract

Acetaminophen (APAP) is perhaps the most commonly used drug both inside and outside the hospital due to its relative safety and over-the-counter availability. Despite its safety, it can cause drug-related side effects, especially acute liver injury that can be unpredictable. Additionally, due to its variable, delayed and nonspecific symptomatology, it can pose a significant diagnostic challenge. Due to potential reversibility with an antidote and adverse outcome related to liver failure, timely recognition and treatment is key in suspected toxicity. Here we present a case of a young female who presented for the evaluation of seizure and found to have APAP-related liver failure with only 2 g of APAP taken over two days duration.

## Introduction

Acetaminophen (APAP) has become the most widely used analgesic-antipyretic in the United States [1]. It is one of the most commonly reported products causing drug-induced liver injury (DILI) [2]. It is also the most common cause of acute liver failure (ALF) in the United States - accounting for 50% of all reported cases and approximately 20% of liver transplant cases [3-4]. The pathophysiology, disease course, and management of ALF secondary to APAP toxicity remain to be precisely elucidated, and adverse patient outcomes with increased morbidity and mortality continue to occur.

The most detrimental clinical presentation is fulminant liver failure, where patients without a history of liver disease present with hepatic encephalopathy and coagulopathy preceding jaundice. Mortality rates have been approximated at 0.4% in overdose patients, translating to 300 deaths annually in the United States. Overall, most cases of APAP toxicity occur after excessive drug intake which can be either intentional or unintentional, a phenomenon frequently referred to in the literature as “therapeutic misadventure.” It is suspected that even repeated therapeutic or slightly excessive doses can be hepatotoxic in susceptible individuals, such as alcoholics [5-6]. Additional reported risk factors include fasting, genetic predisposition, concomitant use of opioids or antiseizure medications, underlying liver disease, old age, and malnutrition [7-9].

Interestingly, APAP toxicity has also been reported even at therapeutic doses without significant concomitant risk factors, which makes us wonder about additional unknown metabolic pathways and yet to be discovered genetic predispositions [10]. APAP hepatotoxicity occurs through the formation of the noxious N-acetyl-para-benzo-quinone imine (NAPQI) metabolite which in turn is augmented by glutathione (GSH) deficiency. N-Acetylcysteine (NAC) is the cornerstone of treatment for APAP toxicity. NAC replenishes and maintains hepatic GSH stores by providing cysteine, the substrate which detoxifies reactive metabolites of APAP [11]. 

## Case presentation

Here we present a case of a young 34-year-old Caucasian female with a history of epilepsy and attention deficit hyperactivity disorder (ADHD), who was brought to the hospital by her boyfriend after a witnessed seizure episode. The patient carries a diagnosis of epilepsy and was recently started on lamotrigine (50 mg twice a day) about one month back. She was also on Adderall for the management of ADHD. At the time of evaluation, she had a poor recollection of the seizure episode but was otherwise at her baseline mentation with no additional complaints. On review of systems, she was admitted having nontraumatic back pain for the past three days. For her back pain, she started taking 1000 mg Tylenol daily, started two days back. She denies having any suicidal ideation or intentional/unintentional drug overdose of any sort. She denies alcohol intake for the past one week and lives with her boyfriend with adequate social support. Review of systems including fever, anorexia, weight loss, nausea, vomiting, diarrhea, or headache was completely negative. She started complaining of moderate abdominal pain while she was in the ER and for which she was given 0.4 mg of intravenous Dilaudid once. Her exam was unremarkable with no abdominal or back tenderness. Her blood work showed new transaminitis with both aspartate aminotransferase (AST) and alanine aminotransferase (ALT) levels of 800 U/L. Her urine drug screen was positive for amphetamines and her serum drug screen showed an APAP level of 47 on admission. No coagulation abnormalities were present and her urine pregnancy test was negative. Her initial Tylenol level was nearly 12 h after the ingestion of the last dose of Tylenol. The next lab work done after 9 h showed worsening transaminitis (AST/ALT of 1000/1200 U/L) along with a rising INR of 1.3. She was upgraded to the medical ICU for N-acetyl cysteine (NAC) treatment and closer monitoring. 

**Figure 1:**
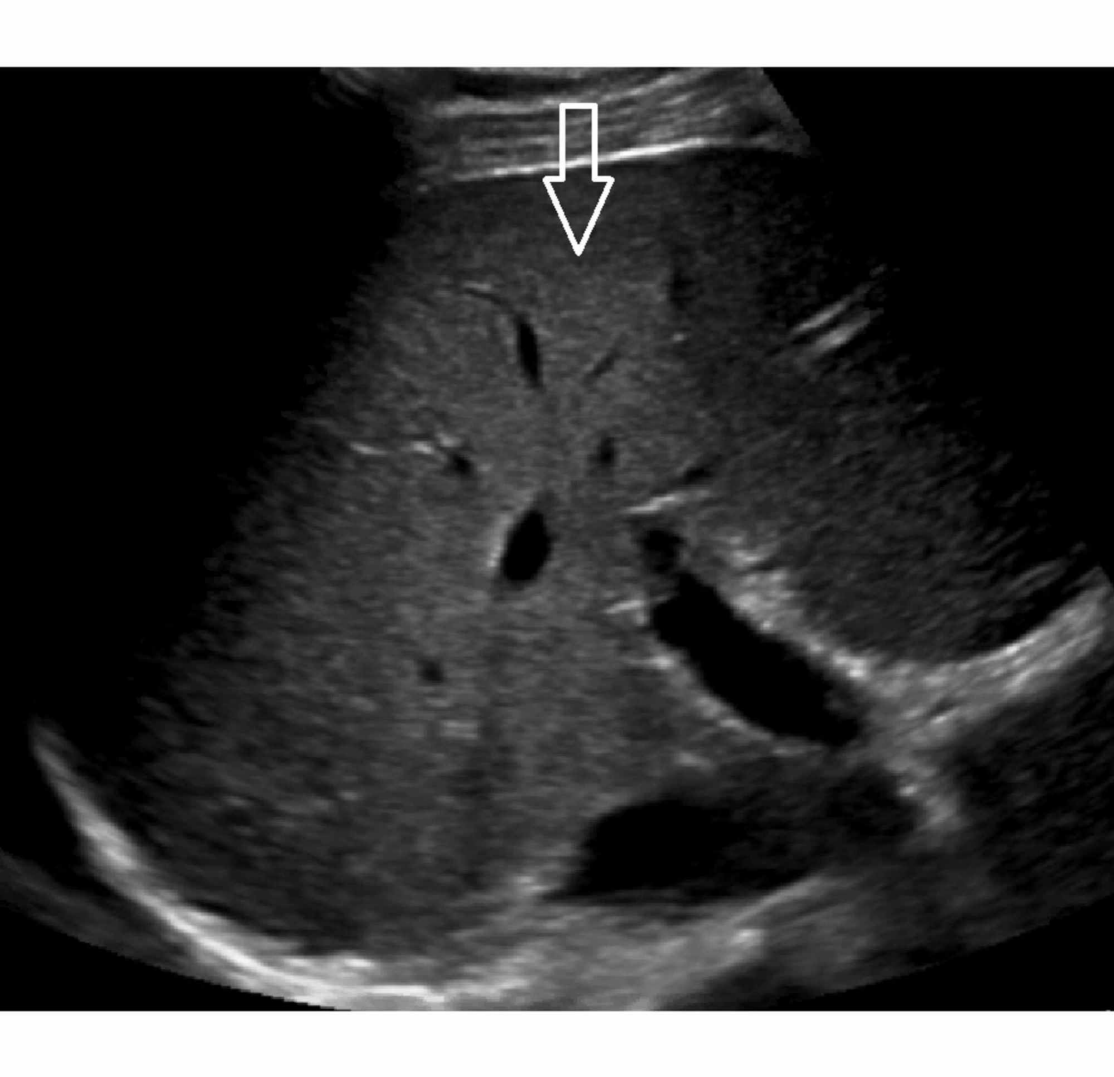
Right upper quadrant ultrasound showing grossly normal liver echo texture (white arrow demonstrating liver).

We initiated NAC treatment after discussing the case with a gastroenterologist and planned to repeat blood work every 12 h along with closer hemodynamic and neurologic monitoring. Meanwhile, additional workup including acute viral hepatitis panel was negative. Right upper quadrant ultrasound showed normal echo texture of the liver (Figure 1).

**Figure 2:**
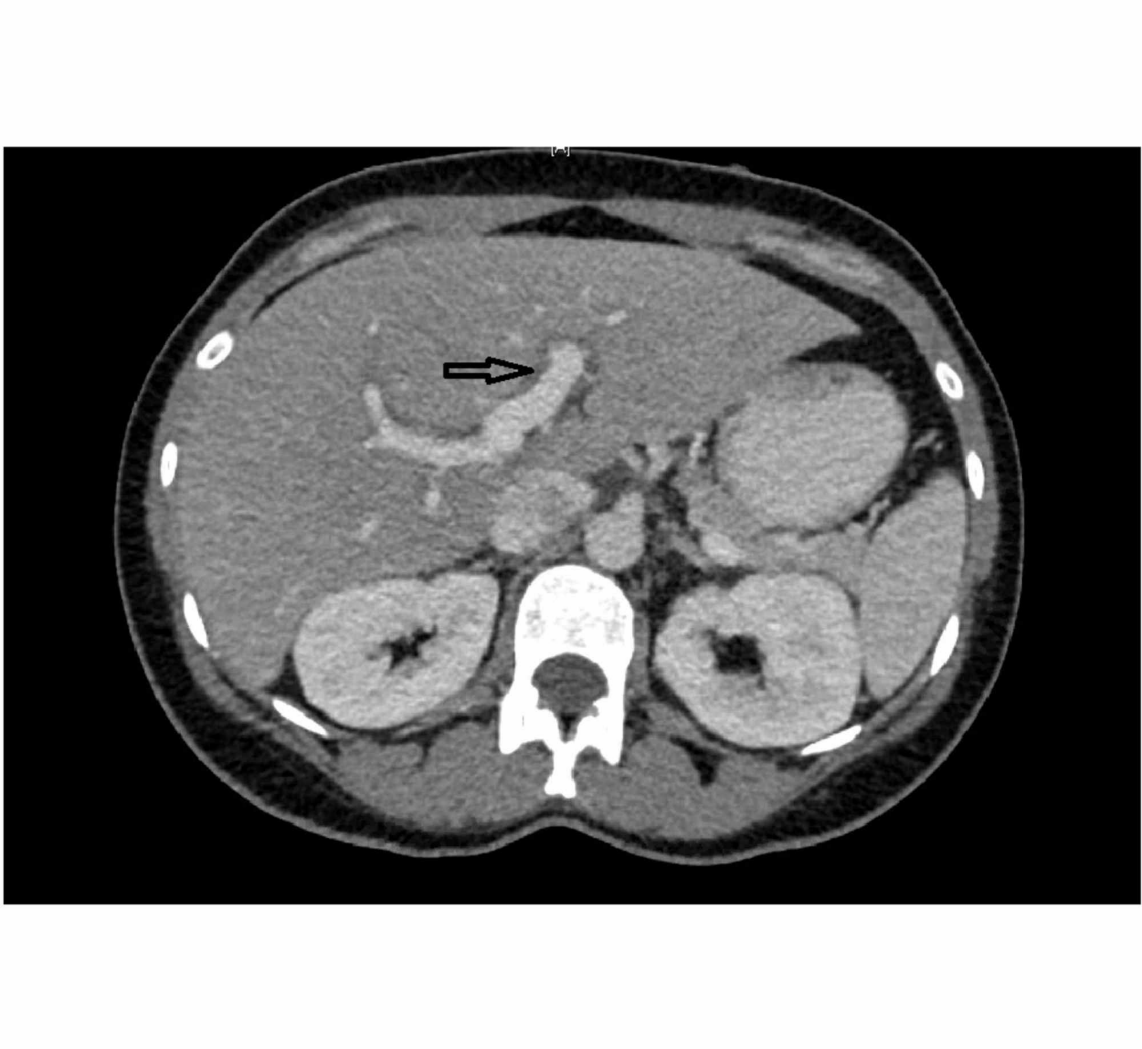
Cross-sectional view of CT scan of abdomen showing peri-portal edema (black arrow).

Repeat blood workup continued to show rising transaminases and next day morning labs showed new hyperbilirubinemia of 2.9 with AST/ALT of 1297/2287. Her INR also rose to 1.8. CT abdomen and pelvis was done and showed mild to moderate periportal edema with no bile duct abnormalities (Figure 2).

Despite treatment with NAC, her liver abnormalities continued to worsen with both AST and ALT levels >5000 and INR of 2.0. At this point, the patient was transferred to a liver transplant center for further care and transplant evaluation.

## Discussion

Acetaminophen toxicity continues to be a challenge both at the diagnostic and management levels. What made this case all the more interesting was the dosage of Tylenol our patient was on. Even though we commonly encounter APAP overdose cases in the hospital, still it is very rare to develop this level of liver dysfunction while being on a minimal dose of APAP. There is literature supporting that concomitant use of drugs or herbal products that induce certain cytochromes like CYP2E1, enzymes can predispose to hepatotoxicity in the absence of overt APAP overdose [12]. This, in turn, may worsen the outcome of an intentional overdose. Examples of medications that alter CYP2E1 activity include certain anticonvulsants like carbamazepine, phenytoin, phenobarbital, and anti-tuberculosis medications like rifampicin and isoniazid [12]. While our patient was recently started on lamotrigine, this medication is not amongst the anticonvulsants that would significantly affect the metabolism of APAP by affecting CYP2E1. It also seems very unlikely that this level of interaction would have occurred at such a low dose of both medications. Additionally, there were no rashes, arthralgias, blood eosinophilia, or any other markers to indicate hypersensitivity reaction to either of these medications. 

While some people exhibit more frequent side effects of APAP toxicity compared to others despite lacking risk factors, which make us wonder about still unknown metabolic pathways or genetic predispositions that account for this response variability. There have been rare case reports regarding late APAP toxicity through idiosyncratic drug reaction via allergic pathways [13]. In that referenced case report, liver toxicity progressed to take the form of chronic hepatitis. Recently more and more data are obtained regarding the use of serum messenger RNAs (miRNAs) as biomarkers of intrinsic and idiosyncratic acute hepatotoxicity and several new genetic and molecular pathways are being worked upon [14].

**Figure 3:**
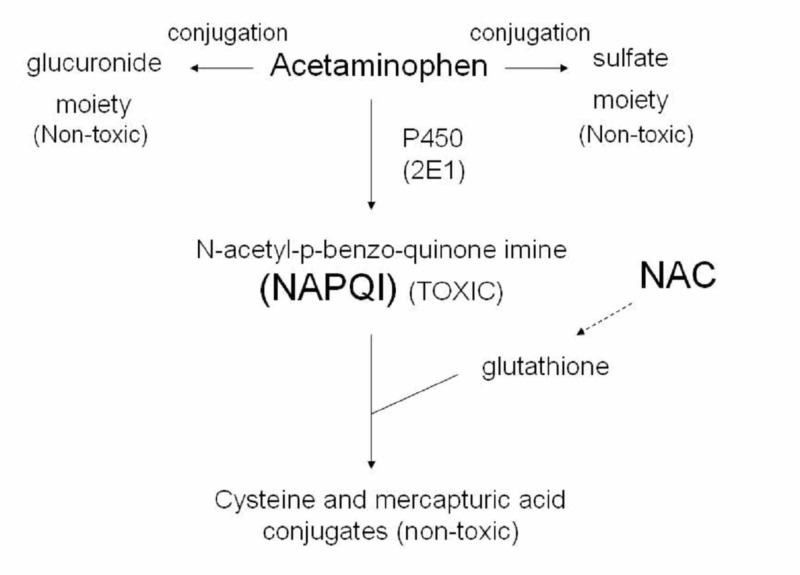
Acetaminophen metabolism and production of toxic metabolite (Courtesy of Wikipedia). Cytochrome P450 (P450), Cytochrome P450 system type 2E1 (2E1), N-Acetyle Cysteine (NAC)

As briefly discussed previously, APAP hepatotoxicity occurs through the formation of the noxious NAPQI metabolite which in turn is augmented by GSH deficiency (Figure 3).

While timely administration of NAC remains the standard of care in APAP toxicity, as discussed above, targeting APAP hepatotoxicity at the molecular level as an alternative to NAC therapy has been the goal of many research groups [15]. Although most patients recover with conservative medical management, early recognition of clinical worsening and timely referral to a liver transplant center remain the most important possible life-saving component of patient management.

Despite the dramatic and rapid worsening of liver function, our patient remained remarkably asymptomatic throughout the hospital stay. She was transferred to a liver transplant center due to rapidly worsening lab abnormalities despite NAC treatment.

## Conclusions

Idiosyncratic Tylenol reaction is a poorly understood but increasingly recognized cause of ALF. This type of reaction is not dose-dependent and high index of suspicion is required to timely diagnose and appropriately treat this type of drug reaction. Due to its rarity, scarce data only in the form of few case reports are available and continued physician education is required to recognize and treat this life-threatening drug reaction. This case presents a form of idiosyncratic drug reaction that unfortunately is not preventable at the moment but extensive research at the molecular level is going on to better understand the additional molecular and genetic pathways involved in it. Additionally, this case encourages us to include serum APAP levels in evaluating a patient with unexplained severe hepatitis/liver failure and we need to be very careful to exclude APAP toxicity based on history only.
